# Glyoxal as Single Crosslinker for Mechanically Blown, Condensed and Hydrolyzable Tannin Foams

**DOI:** 10.3390/polym17223008

**Published:** 2025-11-12

**Authors:** Jonas Eckardt, Michele De Nato, Elena Colusso, Lorenzo Moro, Primož Šket, Samuele Giovando, Gianluca Tondi

**Affiliations:** 1 TESAF Department, University of Padua, Viale dell’Università 16, 35020 Legnaro, Italy; 2 Department of Industrial Engineering, University of Padova, Via Marzolo 9, 35131 Padova, Italy; elena.colusso@unipd.it (E.C.); lorenzo.moro@unipd.it (L.M.); 3 Slovenian NMR Centre, National Institute of Chemistry, Hajdrihova 19, 1000 Ljubljana, Slovenia; 4 Centro Ricerche per la Chimica Fine Srl for Silvateam Spa, Via Torre 7, 12080 San Michele Mondovì, Italy

**Keywords:** chestnut tannin, quebracho tannin, rigid foam, physical properties, hardener, crosslinking mechanism

## Abstract

Tannin foams are polymeric, porous materials produced from plant tannins, with good thermal insulation and fire-retardant properties. Although research has mainly concentrated on usage of condensed tannins (CTs), interest in a second group, hydrolyzable tannins (HTs), is growing. This study evaluated the usability of glyoxal as a single crosslinker for condensed and hydrolyzable tannins in foams created through mechanical agitation, using various ratios of chestnut (HT) and quebracho (CT) tannins. Glyoxal could react with chestnut tannin, but foams with only chestnut collapsed before hardening due to its slow reactivity, with 70% chestnut as the maximum viable content. Increasing the chestnut tannin amount reduced the foamability and compression strength, resulting in higher density and increased pore size. At a similar density (~210 kg m^−3^), the 70%-HT foam reached only one-third the compressive strength of the pure CT foam (0.22 vs. 0.61 MPa), while the pure CT foam showed a smaller mean pore size (189 vs. 365 µm) despite its lower mean density (208 vs. 241 kg m^−3^). The fire resistance and thermal conductivity appeared unaffected by the tannin type and instead depended on the foam density, with thermal conductivities ranging from 56 to 71 mW/(m·K). Leaching tests showed a slight increase in leaching for formulations with higher chestnut tannin contents, with 15% to 24% of acid recovered after the leaching cycle. The ^13^C-NMR analysis revealed the glyoxal crosslinks at the free position of the A-ring in CTs and at the free ortho ones of the gallic/ellagic moieties in HTs. Overall, this study demonstrated that tannin foams can be produced using glyoxal as a single crosslinker, allowing for up to 70% substitution of the condensed tannin component in the formulation.

## 1. Introduction

Tannin foams are bio-based, porous materials that have gained increasing research interest over the past decade as a potential alternative to synthetic foams like polyurethane, EPS and XPS [1,2,3]. Due to their lightweight properties and good fire resistance [4,5], they have been proposed for use as insulation material [6,7,8]. In addition to insulation, they have demonstrated potential in various fields, including adsorption of pollutants in wastewater treatment [9,10], mitigation of ammonia emissions from cattle manure [11,12], use as floral foams [13] and acoustic insulators [14] and for composite sandwich panels [15]. Tannins, which serve as the primary building block that are polymerized and processed into a foam material, are polyphenolic compounds found in plants. They can be chemically categorized into different groups, such as hydrolyzable tannins (HTs) and condensed tannins (CTs), and are already industrially extracted, primarily from various types of trees, for traditional use in the tanning and food industries [16,17,18,19]. While condensed tannins (CTs) have been the focus of tannin foam research [1,2,20], hydrolyzable tannins (HTs) have received less attention due to their lower reactivity, although they have gradually been gaining interest in recent years [21,22,23,24]. Exploring the full potential of various tannin types and crosslinking systems is crucial to developing sustainable materials with diverse applications. The first generation of tannin foams used formaldehyde as a crosslinker in a one-step synthesis under acidic and alkaline conditions [25], but health concerns led to its replacement with furfuryl alcohol, which remains the most popular choice for its good reactivity [6,26,27,28]. It has also been shown that hexamine can act as a sole crosslinker [29,30], while crosslinkers such as glyoxal [31,32], glutaraldehyde [33,34], PVOH [35], humins [36], isocyanates [37] and different proteins [38,39,40] have been explored in combination with furfuryl alcohol or hexamine to obtain a range of material properties, including improved mechanical resistance [37,41]. In addition to these crosslinkers used in a one-step synthesis, another approach based on polyurethane chemistry principles has also been explored for both condensed and hydrolyzable tannins over recent years [21,42]. Within such formulations, the greenest approach is to substitute the polyol with tannin and avoid isocyanate usage, called non-isocyanate polyurethanes (NIPUs). NIPU foams can be produced by reacting tannin’s hydroxyl groups with dimethyl carbonate, followed by reaction of the carbonated tannin with hexamethylenediamine [21,42]. Aside from the various polymerization approaches, different foaming methods have been used for tannin foam production. Mechanical foaming involves the agitation of a tannin-containing solution and the addition of a surfactant [29,43,44], which allows air to be incorporated and a wet, stable foam mass to be formed. This foam can then be cured over time, often with the aid of external heating, such as an oven [22,29,43]. Physical foaming relies on the evaporation of a non-reactive blowing agent at elevated temperatures to create a porous structure [45,46]. Furthermore, in urethane-based tannin chemistry, gaseous reaction byproducts can enable the material to foam [21,47]. The aims of this study were to explore glyoxal as a sole crosslinker for tannin foams and to assess its compatibility with hydrolyzable and condensed tannins. Glyoxal, which can be synthesized from natural materials, has already been used as an additional crosslinker alongside furfuryl alcohol [32,34] or hexamine [30,48], but not exclusively, likely due to its slow reactivity, which is reportedly lower than that of formaldehyde and glutaraldehyde in a mimosa tannin–furanic formulation [34]. The use of a mechanical foaming process, which stabilizes the wet foam mass for a certain time, allows glyoxal’s reactivity to be managed, making it possible to develop foams without additional hardeners or crosslinkers. Various ratios of CT from quebracho and HT from chestnut were further tested to also explore the compatibility of glyoxal with different tannin types. The resulting foams were analyzed for their cell structure, compressive strength, fire resistance, thermal conductivity, leaching resistance and acid recovery. Additionally, solid-state ^13^C-NMR analysis was conducted to investigate the polymeric chemistry involved. This work aims to expand the knowledge on glyoxal as a crosslinker for tannin foam production and to assess its compatibility with condensed and hydrolyzable tannins.

## 2. Materials and Methods

### 2.1. Chemicals and Reagents

Industrial tannin extracts of chestnut (*Castanea sativa*) and quebracho (*Schinopsis balancae*) were provided by Silvateam S.p.A. (S. Michele Mondovì, Italy). Glycerol, ethylene glycol and glyoxal (aq. 40%) were purchased from Thermo-Fisher Scientific (Waltham, MA, USA). Tween80 and sulfuric acid were ordered from Merck (Darmstadt, Germany).

### 2.2. Foam Synthesis

Foams were produced using the mechanical stirrer Velp OHS200 Digital (Usmate, Velate, Italy) with a butterfly stirring head from VMA-Getzmann GmBH (Reichshof, Germany). Tannin was first dissolved in a beaker containing a mixture of water, ethylene glycol, glycerol and glyoxal, added in this sequence while being mechanically stirred at 800 rpm. Next, 32% sulfuric acid was slowly added as the catalyst, followed by the addition of the surfactant Tween80 to the mixture, and the stirring speed was increased to 1200 rpm. Within 15 to 20 min, a stable, wet foam mass developed, which was subsequently transferred into a PTFE-coated mold and cured in a convection oven at 80 °C for 24 h. Three foams were produced in a 10 × 10 × 4 cm mold for the compression, fire and cell analyses, while another foam was produced in a larger 15 × 15 × 7 cm mold for assessing the thermal conductivity. Before cutting the produced foams into shape for the different tests, the outer layer (0.5 cm) was removed, and the samples were conditioned at 20 °C and 65% relative humidity for one week. The weight percentages of the different constituents used in the formulations are detailed in Table 1, of which glyoxal and sulfuric acid were used in a diluted aqueous solution of 40 wt% and 34 wt%, respectively, and which is accounted for in the water component.

The tannin component was varied at four chestnut-to-quebracho tannin ratios: 0:100, 30:70, 50:50 and 70:30, with the numbers 0, 30, 50 and 70 indicating the chestnut tannin percentages.

### 2.3. Density and Porosity

The bulk density (d) was determined by measuring the specimen’s weight and dividing it by its volume. The skeletal and apparent densities were determined by using a helium pycnometer (Anton Paar (Tivoli, Italy), Ultrapyc 3000), which enabled the calculation of both the open and closed porosities of the material. Apparent density (d_a_) measurements were conducted on foam samples at a gas pressure of 18 psi, while the skeletal density (d_s_) was determined after crushing the foams and applying a pressure of 10 psi. All samples were dried at 60 °C for 24 h prior to testing. The total porosity of the foams was calculated according to the equation P = 1 − (d/d_s_), while the open porosity was calculated according to P_o_ = 1 − (d/d_a_). In total, 15 samples per formulation were analyzed.

### 2.4. Cell Measurements

Cell size measurements were taken using a LEICA M80 stereo microscope (Wetzlar, Germany) paired with a LEICA DMC4500 camera and LAS image acquisition software (version 4.13). For each formulation, minimums of 50 length and 100 width measurements were conducted along the growth direction. Following a method previously applied to evaluate tannin foam cells [49,50], the average cell diameter (Cell Ø) was calculated using the formula Cell Ø = (π/4) × D, where D is the mean of 150 measurements. Orthotropicity was determined by dividing the cell length by its width. Images from at least five different samples were evaluated, and outlier measurements of very large or small pores were excluded.

### 2.5. Scanning Electron Microscopy (SEM)

SEM pictures were taken using an ESEM (FEI Quanta 200, Eindhoven, The Netherland) equipped with a backscattered electron detector, operating at 20 kV under low-vacuum conditions. A 1 cm^3^ representative foam sample was cut and mounted on the sample holder with carbon tape.

### 2.6. Solid-State ^13^C NMR

The ^13^C-CP-MAS NMR spectra for the tannin extracts and their washed, polymerized products were obtained using a Bruker Avance NEO 400 MHz NMR spectrometer (Bruker BioSpin, Rheinstetten, Germany) fitted with a 4 mm CP-MAS probe. The sample powders were spun at a frequency of 10 kHz and a temperature of 25 °C. A total of 13,000 scans were collected, with a repetition delay of 3 s.

### 2.7. Compression Test

Ten foam samples from each formulation, with dimensions of 3 × 3 × 2 cm, were tested for compressive strength using a Galdabini Quasar 25 (Galdabini, Cardano-Varese, Italy) following ISO 844:2021 [51]. The compression rate was set to 2 mm/min, with the applied force and corresponding deformation recorded to determine the maximum load before material failure. The compressive strength was calculated by dividing this maximum load by the cross-sectional area of the specimens.

### 2.8. Thermal Conductivity

The thermal conductivity was determined using the Transient Plane Source (TPS) method with the Hot Disk 3500 TPS (Hot Disk AB, Gothenburg, Sweden) apparatus. In this system, a double-spiral-shaped nickel foil sensor insulated with Kapton layers is inserted between two foam samples of identical composition, each measuring 4.5 × 4.5 × 2.5 cm. For each formulation, a single pair was measured five consecutive times, and the mean is reported.

For this experiment, sensor 8563 (radius: 9.863 mm) was used, with the power set at 60 mW and a measurement duration of 320 s. The sensor functions as a resistance thermometer and enables the measurement of the temperature increase on the active surfaces of samples over time, which is caused by the applied current. Several studies [52,53] provide detailed descriptions of this technique, which allows for the calculation of the thermal conductivity of foams in mW/(m·K). Measurements were carried out under dry conditions at room temperature (~293 ± 2 K).

### 2.9. Leaching Resistance and Acid Recovery

The leaching resistances of the various formulations were tested in water by drying 4 g of pulverized foam powder at 80 °C, stirring it in 200 mL of aqueous solution for 2 h and then filtering the mixture using a 25 μm paper filter. The filtered powder was dried and weighed to calculate the percentage of material leached. To determine the recovered-acid amount, the solution’s pH was measured using the instrument Medidor PH BASIC 20 (CRISON, Barcelona, Spain), and the sulfuric acid recovery was indirectly calculated from the pH, considering the maximum possible H+ concentration in 4 g of the polymer. The process was repeated three times for each foam formulation.

### 2.10. Fire Resistance

Fire resistance was assessed using a method previously used for tannin foams [49]. Foam cubes with a side length of 2 cm each were placed on a metal grid located on a laboratory stand, which was placed on an analytical balance. The samples were then exposed to a Bunsen burner flame at a distance of 5 cm for 3 min. The weight of each sample was measured every 15 s during the flame exposure and again once the weight stabilized again. Five samples from each formulation were tested, and the average percentage of mass loss was calculated for comparison across formulations.

### 2.11. Statistical Analysis

Statistical analyses were performed in SPSS v27 (IBM, Armonk, NY, USA). Multiple regression models were tested, using the tannin type and other measured material properties as predictors. Significance was set at *p* < 0.05.

## 3. Results and Discussion

Prior to presenting the results, key pretrial observations should be mentioned. Formulations with 100% chestnut tannin consistently collapsed due to the prolonged curing time, a problem worsened with the increased water content in the formulation. Increasing the glyoxal content also did not prevent foam collapse in 100% of the chestnut formulations. Initial tests with quebracho tannin showed that higher glyoxal levels enhanced the mechanical strength, but a lower concentration was chosen for the here-presented foams to maximize the tannin content. Attempts to produce lower-density foams (~150 kg/m^3^) through secondary water addition after 5–10 min were successful but resulted in highly fragile materials for formulations with high chestnut tannin contents. Consequently, a target density of approximately 200 kg/m^3^ was chosen to facilitate a more reliable comparison of the tannin component’s influence.

### 3.1. Foam Structure and Morphology

**Figure 1:**
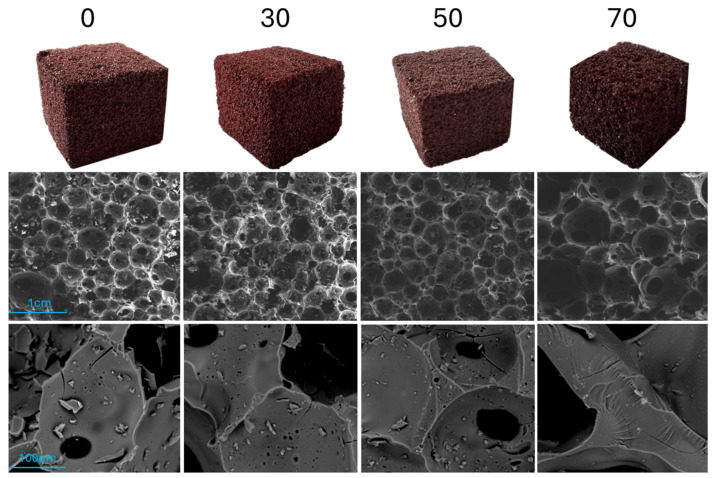
Morphological appearances of different foam formulations from 0 to 70% chestnut tannin content (**left** to **right**).

The foams produced for the comparison of the tannin component’s influence in this study are presented in Figure 1, alongside their SEM images at varying magnifications.

The non-magnified view already suggests that increasing the chestnut content leads to a larger average cell size, which becomes more evident in the SEM images and is confirmed by the cell size measurements, reported in Table 2. While the chestnut component has only a small impact on the cell size and shape up to 50% content, a higher concentration of 70% significantly increases the pore size, resulting in larger, more irregular pores and a less homogeneous structure. Despite these changes, the overall cell structure remains highly interconnected, with a similar pore morphology up to 50% chestnut content. Comparing the cell walls, the 70% chestnut foam showed signs of partial material collapse during curing. This foam also exhibits a notable increase in orthotropicity (Table 2), likely due to the partial collapse. In comparison to tannin–furanic foams of similar density, the quebracho tannin glyoxal foams display slightly larger cell sizes with comparable orthotropicity [22]. Although the high-percentage chestnut tannin foam showed a significant increase in cell size, this effect was not observed in a similar study comparing chestnut and quebracho tannin foams using furfuryl alcohol as the crosslinker [22]. Additional porosity characteristics are provided in Table 2, showing that the total porosity across all formulations was approximately 85%, with the majority consisting of open pores.

The skeletal densities were comparable across formulations, with the highest value for chestnut tannin and the lowest for quebracho tannin. Despite having a porosity very comparable to furfuryl alcohol-based mimosa foams made via mechanical agitation, the bulk densities of the foams in this study were higher due to their higher skeletal densities [43].

### 3.2. Chemical Characterization

**Figure 2:**
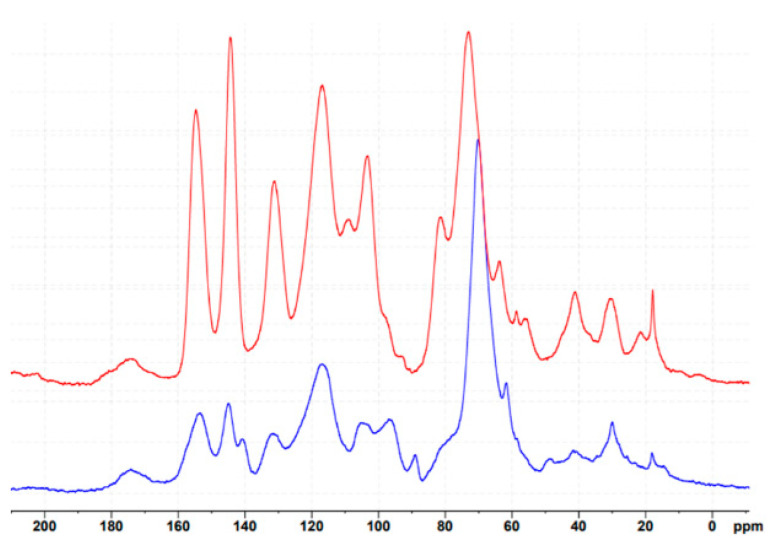
^13^C-NMR spectra of quebracho tannin extract (red) and reacted foam polymer (blue).

While numerous studies have utilized glyoxal as a crosslinker for tannin and lignin in wood adhesives and polymers and as an additive in various tannin foam formulations, the chemical principles governing its crosslinking mechanisms with different tannins are still only partially understood, with variations observed under different reaction conditions [32,54,55,56,57,58]. To further elucidate these mechanisms, solid-state ^13^C NMR analysis was conducted on the tannin extract, and the water-leached foam polymer and corresponding spectra are shown in Figure 2.

**Figure 3:**
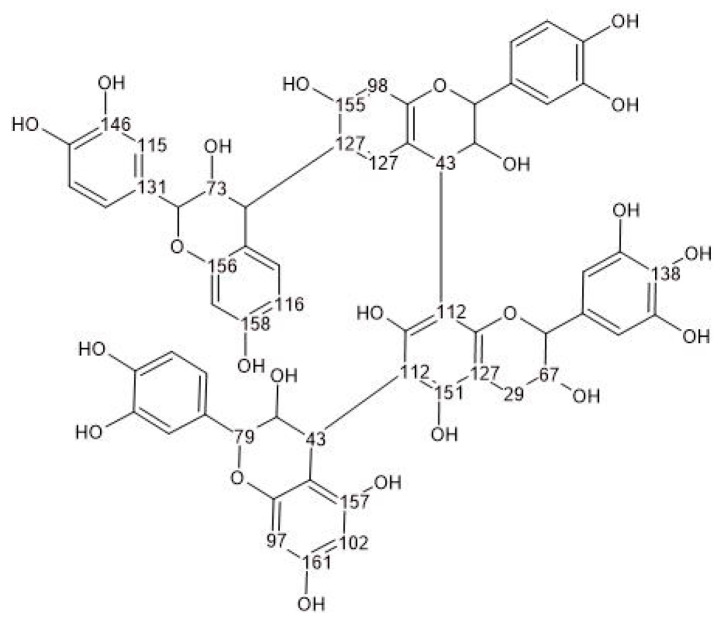
Hypothetical occurrence of monomeric units in quebracho tannin together with its calculated ^13^C-NMR chemical shifts (nmrdb.org [63]).

The spectrum of quebracho tannin (red) shows typical chemical shifts in the range of 160 to 100 ppm. The signals from 165 to 145 ppm correspond to hydroxyl-containing carbons in the A- and B-rings, while the signals around 116 ppm and 97–103 ppm are attributed to free positions on the phenolic A-ring. The 116 ppm signal also corresponds to a hydroxyl-free carbon in the phenolic B-ring. Additionally, the signal at 110 ppm corresponding to the interflavonoid linkage is observed in this region [59]. A proposed chemical structure of quebracho tannin, as suggested by various studies [60,61,62], is presented in Figure 3, along with the calculated chemical shifts using the NMR prediction tool nmrdb.org [63].

**Figure 4:**
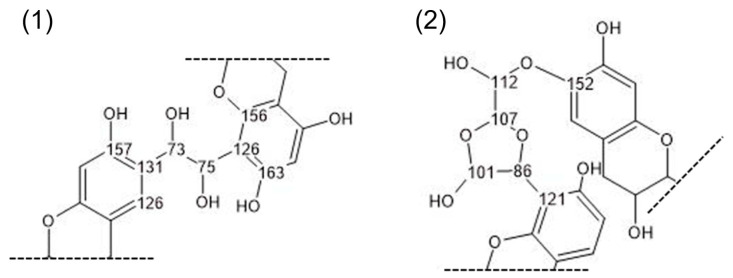
Hypothetical crosslinking mechanism of glyoxal and quebracho tannins. (**1**) Dihydroxy-ethylene bridge; (**2**) dioxolane bridge.

A comparison between the tannin extract and the reacted polymer reveals significant differences in the 165 to 100 ppm region. Interpreting this region is challenging due to signal overlap from both newly formed and pre-existing bonds, which generate similar chemical shifts. Additionally, glyoxal’s tendency to rearrange in aqueous media further complicates the identification of a clear reaction mechanism [64]. The overall signal reduction in the polymer at 116 and 103 ppm compared to the tannin extract suggests an aromatic substitution at the C6 and C8 positions of the A-ring. The signal at 98 ppm that also belongs to a hydroxyl-free carbon in the phenolic A-ring though appears rather increased, likely due to novel glyoxal-related structures and their overlapping signals, but a slight shift of the signal also suggests a reaction on the free position of the A-ring. Although the signal at 115–120 ppm is reduced compared to the tannin extract, it becomes more prominent relative to the signals between 145 and 160 ppm in the polymer, which correspond to hydroxyl-containing carbons in the phenolic ring and are expected to remain unchanged. The relative increase in and broadening of the signal around 117 ppm are likely due to signal overlap from various glyoxal crosslinking formations at the C6 and C8 positions of the phenolic A-ring. Also, the signal around 70 ppm shows a significant increase in the polymer compared to the region of 145 to 160 ppm and can be attributed to various glyoxal crosslinking formations, as well as to residues from tween, glycerol and ethylene glycol. Similarly, the relative increase at 31 ppm could indicate the formation of ethylene bridges (R-CH_2_-CH_2_-R) or traces of tween. A new signal also appears in the polymer spectra at 88 ppm, likely resulting from glyoxal formation, as shown in Figure 4/part 2. While it is difficult to pinpoint a detailed reaction from the observed signals, multiple potential reactions at the free positions of the phenolic A-ring can be hypothesized based on the obtained spectra. Figure 4 illustrates two of these potential reaction products.

While the previous literature suggested different reaction mechanisms of condensed tannins with glyoxal, we could not find similar reactions to those described before due to the absent signals of enol (–CH=C(OH)–) (calc. 184 ppm) and C=O (205 ppm) formation [58].

**Figure 5:**
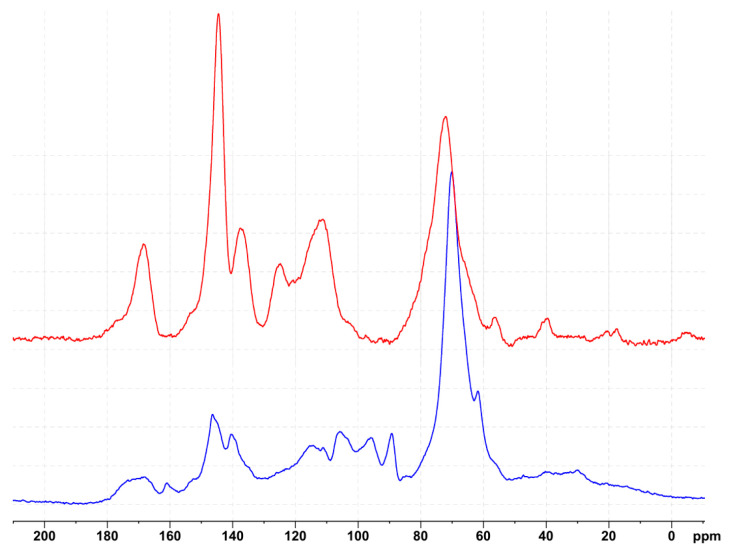
^13^C-NMR spectra of chestnut tannin extract (red) and reacted foam polymer (blue).

The spectra of the chestnut tannin and its reacted and leached foam polymer are reported in Figure 5.

**Figure 6:**
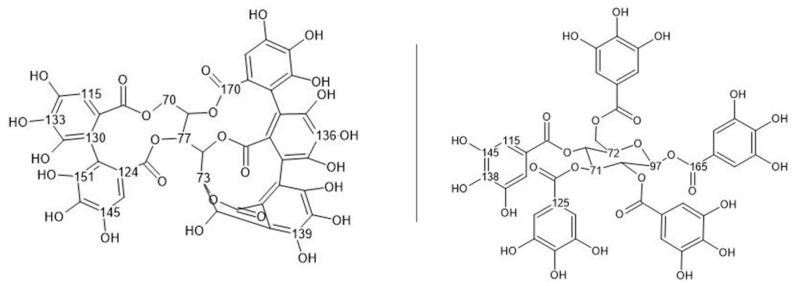
Chemical structures of vescalagin (**left**) and pentagalloylglucose (**right**) as representative compounds of chestnut tannin together with their calculated ^13^C NMR shifts [63].

Chestnut tannin is a diverse mixture of polyphenolic compounds, composed mainly of ellagic acid derivatives such as castalagin and vescalagin, followed by gallic acid and its derivatives, known as gallotannins [65,66,67,68,69]. Figure 6 shows the chemical structures of an ellagitannin (vescalagin) and a gallotannin (pentagalloylglucose), representative of the primary constituents in chestnut tannin, to aid in understanding the reaction mechanisms involved.

**Figure 7:**
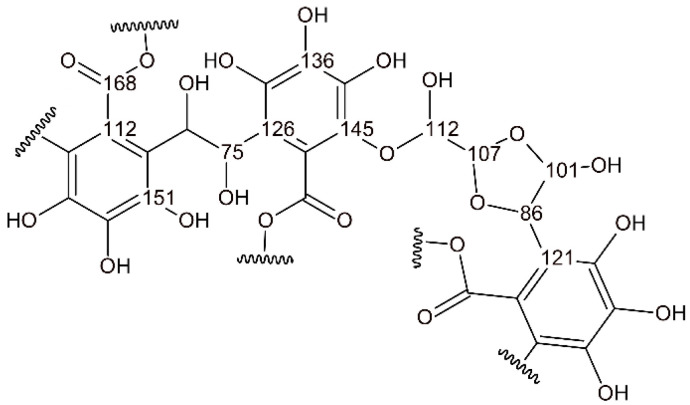
Possible simplified reaction mechanism of chestnut tannin with glyoxal.

From these model structures, several conclusions can be drawn about the spectral differences between unreacted and reacted chestnut tannin. The 130–155 ppm region corresponds to the aromatic carbons holding an -OH group, which differs between the spectra of the reactant and pure tannin extract. In the example of gallic acid, the two carbons in the meta position resonate at 145 ppm, while those in the para position resonate at 138 ppm. When the phenolic unit in chestnut is polymerized by the direct substitution of the ortho position, the signal from the meta position at 145 can be observed in slightly higher fields (towards 151 ppm). Similarly, the signal around 165 of the carboxyl carbon that broadens and shifts towards 175 indicates a crosslinking reaction taking place in the ortho position. The reaction at this position is also highlighted by the change and reduction in the signal at 115 ppm, which corresponds to the free aromatic carbon itself. However, interpretation becomes more complex when considering the relative intensities of the signals in the region of the more stable hydroxyl-keeping carbons (130–155 ppm). This is further complicated by the broadening and overlapping of several signals around 110 to 126 in the reacted spectra, which can be explained by the proposed reaction mechanism shown in Figure 7 together with its calculated chemical shifts.

Based on this reaction mechanism, the previously free ortho position, typically observed around 115 ppm, overlaps with the shifted signal of C1, which moves from 124 ppm to 112 ppm after crosslinking. Additionally, newly formed glyoxal bonds contribute signals in the same region. Furthermore, the disappearance of the C1 signal around 125 ppm after crosslinking, followed by its reappearance in the carbon of the previously free ortho position at 121 and 126 ppm when reacted, complicates interpretation of the signals in this area. Other differences in the reacted chestnut spectrum are the appearances of new signals at around 105 and 90 ppm, which can be attributed to the crosslinking structure of glyoxal reported in Figure 7. Further, the strong increase in the region of 70 ppm potentially comes from the suggested reaction mechanism of glyoxal and traces of ethylene glycol, glycerol and tween. In summary, the polymerization of chestnut with glyoxal likely follows a similar pattern to that observed in the polymerization of quebracho with glyoxal, involving the binding of glyoxal moieties at the free ortho positions of the aromatic ring. Due to the higher reactivity of the ortho and para positions of the phenolic rings in the flavonoid, the number of crosslink bridges is expected to be higher for CTs than that occurring with gallic moieties (HTs), where the -OH is also in the meta position, reducing the availability of hydrogen for the aromatic electrophilic substitution.

### 3.3. Functional Properties

**Figure 8:**
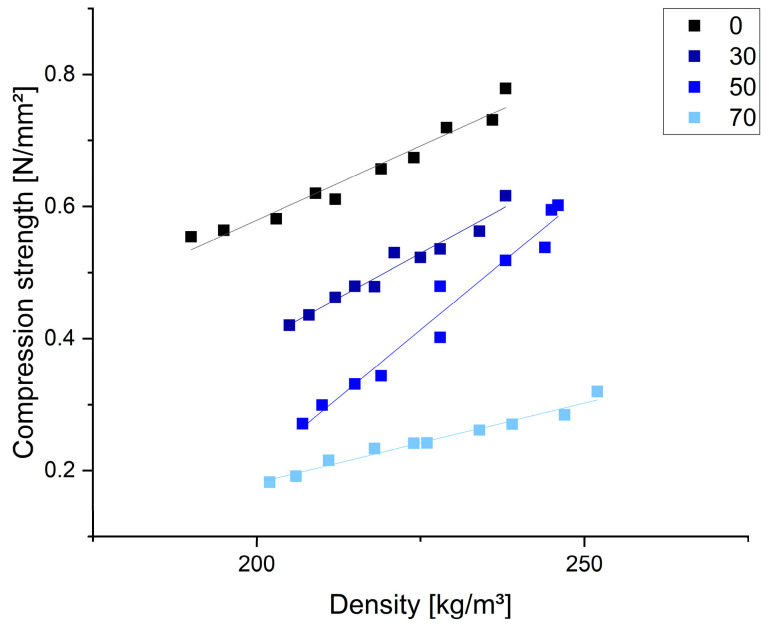
Compression resistances of different tested samples according to their densities and chestnut tannin amounts (0, 30, 50, 70).

The results of the compression tests are reported against the sample densities in Figure 8.

A clear correlation between the density and compression resistance was observed within each formulation, with significant differences when the chestnut tannin content was varied. As the proportion of chestnut tannin increases, the material’s compression strength decreases. Aside from differences in the cellular structure, easier activation of condensed flavonoid units (CTs) compared to HTs may increase crosslinking, yielding higher mechanical strength. The quebracho-based formulations showed compression strengths comparable to those of tannin–furanic foams of similar density produced by using diethyl ether as the blowing agent [70]. However, when compared to mechanically blown mimosa tannin–furanic foams [43], the foams produced in this study demonstrate lower compression resistance (0.55 N/mm^2^ at 190 kg/m^3^ compared to 0.84 N/mm^2^ at 170 kg/m^3^) [43]. This difference may be due not only to the tannin type but also to the crosslinker content, which was significantly lower in the present formulations and had already been shown in pretrials to enhance the material strength. The 70% chestnut tannin formulation showed significantly lower compression strength compared to that of other chestnut tannin–furanic foams reported in the literature [22], but they remained comparable to chestnut tannin foams produced using non-isocyanate polyurethane (NIPU) chemistry [23]. The results of the thermal conductivity measurements for the different formulations are shown in Table 3 together with their materials’ densities.

The thermal conductivities registered were between 56 and 71 mW/(m·K), with the lowest being the 0% chestnut formulation and the highest being the 70% chestnut formulation.

While the sample size was too small to detect statistical differences between the different tannin types, it appears that the determining factor is their density, which has also been shown in previous studies to be the major factor influencing the thermal conductivity of tannin foams [45,71]. Compared to other tannin foams with similar densities produced via mechanical agitation [22,43], the herein-produced foams tendentially show higher thermal conductivity. This difference could be attributed not only to variations in the pore structure but also to the differing skeletal densities. Overall, their thermal conductivity values align well with those reported for tannin foams of comparable densities [45,70,71,72].

**Figure 9:**
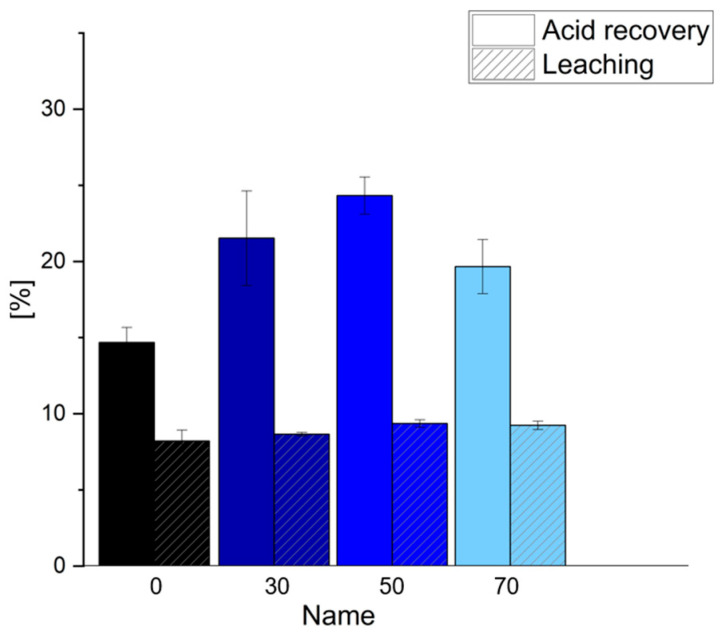
Percentages of leached material and recovered acid for different formulations of 0 to 70% chestnut tannin.

The leaching resistance against water was measured for each formulation after pulverizing the foams, and the average results of the leached amounts for the different formulations are displayed in Figure 9, together with the amount of the recovered-acid catalyst.

The leached material is very comparable across the formulations, ranging from 8 to 9%, with the lowest value for the pure quebracho formulation and the highest for the 70% chestnut formulation. These values are lower than those reported for tannin–furanic foams in a previous study (14–19%) [22] but align with levels found in other tannin–furanic research [43]. Leaching can vary not only due to incomplete polymerization but also because of non-polymerized components in the formulation, such as plasticizers, which can be leached by water. Though leaching differences between formulations were minor, prior studies found that chestnut-based furanic foams were significantly more prone to leaching than quebracho-based foams [22]. Despite the high proportion of potentially leachable components, the low values suggest that some materials, such as glycerol, ethylene glycol or Tween80, remain partly trapped within the polymer. Acid recovery ranged from 15 to 24% and was highest for the 50% formulation and lowest for the 0% formulation. This tendency can also be explained by the more crosslinked quebracho polymer, which embeds the catalyst inside the structure. These values are comparable to those for tannin–furanic foams in previous studies [22] and show that only a limited amount of acid is recoverable with the chosen washing cycle.

**Figure 10:**
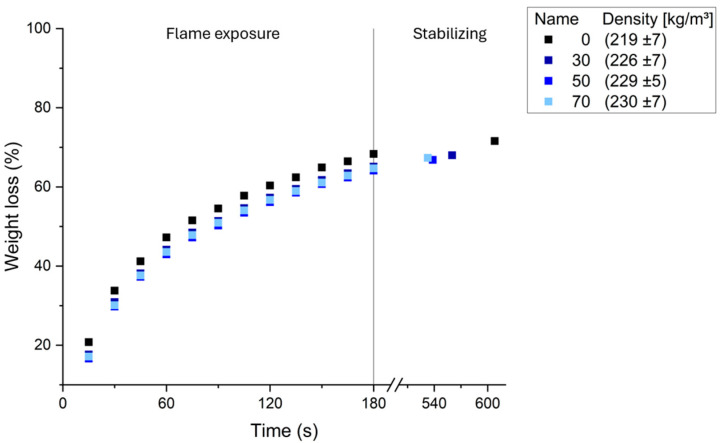
Average burning behaviors of different formulations.

The average burning behaviors of the five tested samples are presented in Figure 10 for the different formulations.

All formulations exhibited similar burning patterns, characterized by a rapid weight loss of approximately 45% within the first 60 s, followed by a gradual decrease in the burning rate, resulting in a total weight loss between 64% and 68% by the time the flame was turned off. After the Bunsen burner was turned off, the foams self-extinguished within 5 to 10 s and continued to lose an additional 3% of weight for approximately 6 min before stabilizing. Among the different formulations, the 0% formulation exhibited the highest overall weight loss, while the 50% formulation had the lowest. Although the tannin content appeared to affect the final weight loss, an ANCOVA, with density as a covariate and formulation as a factor, revealed no significant effect of the tannin type on the weight loss, with only density showing a significant influence (*p* < 0.001, R^2^ = 0.865). These weight loss values observed are comparable to those for tannin–furanic foams [49].

## 4. Conclusions

This study evaluated glyoxal’s effectiveness as a crosslinker for condensed and hydrolyzable tannins in foams created through mechanical agitation, using various ratios of chestnut and quebracho tannins. While glyoxal could react with quebracho tannin, formulations containing only chestnut tannin failed due to the slow curing reaction, causing foam collapse before hardening. The highest feasible chestnut content without notable cell collapse was 70%. Increasing the chestnut content reduced the foamability, leading to higher density and greater cell size. Foams with 70% chestnut displayed significantly larger, more irregular pores and partial cell wall collapse during curing, while foams with lower chestnut contents retained a more consistent morphology. Mechanical testing revealed that as the proportion of chestnut tannin increased, the compression strength of the foams decreased, and that within a formulation, a clear correlation between density and compression strength was found. The thermal conductivity measurements ranged from 56 to 71 mW/(m·K), primarily influenced by the material density rather than the tannin type.

Leaching tests indicated that formulations with higher chestnut tannin contents had slightly increased leaching and acid recovery, ranging from 15% to 24%. The different foam formulations exhibited similar burning profiles, with the total weight loss ranging from 64% to 68%, primarily influenced by the density and not by the type of tannin.

The chemical investigation through solid-state ^13^C-NMR analysis indicated the aromatic substitution of the glyoxal and the hydrogens of the free positions on the phenolic A-ring of the quebracho tannin, likely through dihydroxylethylenic bridges and more complex heterocyclic adducts from glyoxal curing, which were also highlighted in the spectra of chestnut–glyoxal polymers. In the latter, the binding positions are the free ortho- positions of the gallic/ellagic moieties. Independently of cell structure differences, the higher reactivity of condensed flavonoid units may lead to a higher crosslink density and improved mechanical strength.

Overall, this study demonstrated that glyoxal can effectively serve as a single crosslinker in condensed tannin foams produced by mechanical agitation, allowing up to 70% substitution with chestnut tannin while still yielding viable foam materials, and up to 50% without significant changes in the foam morphology. However, higher levels of chestnut tannin in the formulation led to decreased compression strength and foamability, while the fire resistance and thermal conductivity were unaffected by the tannin type and instead depended on the foam density.

## Figures and Tables

**Table 1:** Basic formulation for substituting different amounts of quebracho by chestnut tannin.

Tannin [%]	Water [%]	Glyoxal [%]	Ethylene Glycol [%]	Glycerol [%]	Sulfuric Acid [%]	Tween80 [%]
39.6	30.7	9.5	5	3.6	3	8.6

**Table 2:** Results of porosity, density and cell size measurements with standard deviations reported in parentheses.

Name	Bulk Density [kg/m^3^]	Skeletal Density [kg/m^3^]	Total Porosity [%]	Open Porosity [%]	Calc. Cell Diameter [µm]	Orthotropicity
0	208 (23)	1431 (2)	85.5 (1.6)	83.3 (2)	189 (65)	1.17 (0.30)
30	216 (3)	1442 (2)	85 (0.2)	82.7 (0.2)	247 (74)	1.21 (0.23)
50	220 (2)	1444 (2)	84.8 (0.1)	83 (0.9)	261 (94)	1.21 (0.30)
70	241 (30)	1454 (3)	83.4 (2.1)	82.9 (2.5)	365 (126)	1.25 (0.30)

**Table 3:** Results of the thermal conductivity measurements for the different formulations of 0 to 70% chestnut tannin (standard deviations in parentheses).

Name	Density [kg/m^3^]	Thermal Conductivity [mW/(m·K)]
0	190 (1.61)	56.4 (0.2)
30	225 (2.15)	68.4 (0.4)
50	210 (3.49)	67.4 (0.3)
70	300 (5.12)	71.2 (0.3)

## Data Availability

The original contributions presented in this study are included in the article. Further inquiries can be directed to the corresponding author.
